# PVA-Based Mixed Matrix Membranes Comprising ZSM-5 for Cations Separation

**DOI:** 10.3390/membranes10060114

**Published:** 2020-05-30

**Authors:** Fangmeng Sheng, Noor Ul Afsar, Yanran Zhu, Liang Ge, Tongwen Xu

**Affiliations:** 1 CAS Key Laboratory of Soft Matter Chemistry, iCHEM (Collaborative Innovation Center of Chemistry for Energy Materials), Department of Applied Chemistry, School of Chemistry and Materials Science, University of Science and Technology of China, Hefei 230026, China; sa052@mail.ustc.edu.cn (F.S.); noor@mail.ustc.edu.cn (N.U.A.); zhuyr@mail.ustc.edu.cn (Y.Z.); 2 Applied Engineering Technology Research Center for Functional Membranes, Institute of Advanced Technology, University of Science and Technology of China, Hefei 230088, China

**Keywords:** ZSM-5 zeolite, electrodialysis, monovalent cation separation, mixed matrix membrane

## Abstract

The traditional ion-exchange membranes face the trade-off effect between the ion flux and perm-selectivity, which limits their application for selective ion separation. Herein, we amalgamated various amounts of the ZSM-5 with the polyvinyl alcohol as ions transport pathways to improve the permeability of monovalent cations and exclusively reject the divalent cations. The highest contents of ZSM-5 in the mixed matrix membranes (MMMs) can be extended up to 60 wt% while the MMMs with optimized content (50 wt%) achieved high perm-selectivity of 34.4 and 3.7 for H^+^/Zn^2+^ and Li^+^/Mg^2+^ systems, respectively. The obtained results are high in comparison with the commercial CSO membrane. The presence of cationic exchange sites in the ZSM-5 initiated the fast transport of proton, while the microporous crystalline morphology restricted the active transport of larger hydrated cations from the solutions. Moreover, the participating sites and porosity of ZSM-5 granted continuous channels for ions electromigration in order to give high limiting current density to the MMMs. The SEM analysis further exhibited that using ZSM-5 as conventional fillers, gave a uniform and homogenous formation to the membranes. However, the optimized amount of fillers and the assortment of a proper dispersion phase are two critical aspects and must be considered to avoid defects and agglomeration of these enhancers during the formation of membranes.

## 1. Introduction

Electrodialysis (ED) based on ion-exchange membranes (IEMs) is an important separation technology, which has been widely used for seawater/brackish water desalination, wastewater treatment, acid-base recovery, selective ion separation due to the low energy consumption, no phase transition, and high productivity [1,2,3,4,5,6,7]. With the development of innovative IEMs, the membrane-based separation techniques have expanded further and played a crucial role in energy-saving and clean production [8,9,10,11,12]. The conventional IEMs can only separate oppositely charged ions (Donnan effect). However, it may not separate ions with the same charge [5]. The best example can be observed for the selective removal of ions from the industrial waste-acid solution containing Cu^2+^, Zn^2+^, Ni^2+^, and other heavy metal ions that require further acid recovery and extraction of heavy metal ions to prevent environmental pollution and to realize the utilization of resources trapped in the wastewaters [13,14]. Similarly, the enrichment of Li^+^ ions and the production of edible salt (NaCl) from brines [15,16] are also critical challenges for the traditional IEMs to overcome. Monovalent cations are always accompanied by the divalent cations such as Mg^2+^ and must be removed selectively to circumvent membranes scaling in the ED process. The scaling may decrease the ion flux and the process efficiency [17,18,19,20]. Therefore, it is urgently needed to develop IEMs with unique features to separate monovalent ions from the divalent ions for desired applications. Recently, various researchers employed monovalent ion selective membranes to overwhelm the problems mentioned earlier. For example, Zhang et al. proposed the idea of selectrodialysis to separate monovalent ions from divalent ions [21]. A few studies also show the selective separation of Li^+^ from Mg^2+^ by ED with the monovalent ion selective membranes [22,23]. In fact, the commercial monovalent ion selective membranes are expensive and show low permselectivity. In addition, the high area resistance and low limiting current density restrict their industrial applications. Therefore, we focused on developing a facile way to prepare selective membranes for monovalent/divalent cations separation.

The permselectivity of the traditional IEMs could be improved in multiple approaches: (i) crosslinking to get high density of the membranes’ structure, [24] (ii) membranes surface coating with a positively charged layer to facilitate the exclusion of multivalent cations via electrostatic repulsion, [23,25] and (iii) blending of polymers with either other polymers or microporous crystalline materials such as zeolites to systematize the permselectivity of mixed matrix membranes (MMMs) [26,27]. Zeolites are crystalline aluminosilicates with systematic pores and cavities of molecular dimensions and are famous for their excellent ion-exchange capacity [28]. Zeolites are formed by interlinked tetrahedral of SiO_4_ and AlO_4_^−^ sharing an oxygen atom with a large number of exchangeable cationic sites at AlO_4_^−^. These sites could be easily exchanged by Li^+^ and H^+^ and afford excellent ion conductivity [29,30,31]. Ample surface area, strong adsorption capacity, and molecular separation make zeolites conducive for catalysis, heavy metals ions adsorption, and other separation applications [32,33,34]. 

Due to the tunable porosity of zeolites, it has been used for ion separation from aqueous solution. For example, Dong et al. [35] introduced NaY zeolite nanoparticles in the polyamide interfacial polymerization to increase the salt rejection up to 98.8%. Similarly, Fathizadeh et al. [36] doped NaX zeolite into the polyamide surface layer to prepare MMMs with enhanced surface properties, such as contact angle, surface roughness, and solid-liquid interface free energy. The reported MMMs showed high water flux, which was 1.8 times higher as compared with the polyamide membrane without zeolite. In addition, ZSM-35 zeolite was also used to construct a thin layer on the poly (ether sulfone) (PES) porous membrane using Nafion solution as a crosslinking agent [31]. With the help of a 0.5 nm pore size, the resultant membrane showed excellent permselectivity for hydrated proton (<0.24 nm) and vanadium ions (0.6 nm). Polysulfone (PS) and zeolite-based membranes have been reported for efficient adsorption of Cu^2+^ ions from aqueous solution [37]. Except for ions permselectivity, various MMMs have been recently investigated for additional separation applications [38,39]. In summary, due to the high permselectivity, dimensional stability, low cost, and easy preparation of membranes, MMMs originated as an exciting theme for modern research. Inspired by the well-crystalline and microporous structure of the zeolite, we prefer to amalgamate this nano stuff inside the PVA polymer for selective ions separation.

In the present study, commercial ZSM-5 zeolite of low price with an appropriate pore size (0.5–0.6 nm) was used to prepare ZSM-5/PVA-based MMMs by simple mixing and a casting procedure. Due to the sieving effect and cationic exchange sites of the ZSM-5 zeolite, we developed low-cost MMMs with various contents of zeolite in the PVA backbone for selective separation of monovalent cations via the ED process. Additionally, the influence of zeolite contents was evaluated to optimize the dosage of zeolite for the membrane’s synthesis. The physicochemical properties, such as microstructures analysis, water uptake (WU), area swelling, and membrane’s resistance, were examined and explained in detail. The prepared MMMs were investigated for the H^+^/Zn^2+^ and Li^+^/Mg^2+^ systems, respectively.

## 2. Experimental

### 2.1. Materials

ZSM-5 zeolite was purchased from Shentan Environmental Protection New Materials Co. Ltd. (Shanghai, China). Polyvinyl alcohol (PVA) was obtained from Shanghai Macklin Biochemical Co. Ltd. (Shanghai, China). Commercial anion exchange membranes AMX (Neosepta, Tokuyama Co., Tokyo, Japan) were used as auxiliary membranes in the ED experiments. Other reagents, including LiCl, MgCl_2_, ZnCl_2_, HCl, NaOH, and dimethyl sulfoxide (DMSO), were supplied by China National Pharmaceutical Group Industry Co. Ltd. (Beijing, China). All these reagents were of an analytical grade and used without any further purification. Deionized (DI) water was used in all experiments.

### 2.2. Preparation of ZSM-5/PVA-Based MMMs

PVA (10 wt%) transparent solution was prepared in the DMSO at 100 °C for 1.5 h. Thereafter, the PVA solution was cooled down to room temperature and stabilized for a certain time. The membrane casting solutions were prepared by mixing various amounts of the ZSM-5 in the PVA solution, and the corresponding membranes were named x-ZSM-5 (x = 20, 30, 40, 50, and 60 in weight percent). The mixed solutions were then magnetically stirred and sonicated for 30 mins to get homogenous solutions. Lastly, the resultant mixed solutions were cast on a clean glass plate at 60 °C for 12 h. The membranes were peeled off and hydrated with 0.1 mol L^−1^ solution of HCl for further use.

### 2.3. Characterization of ZSM-5/PVA-Based MMMs

The surface and cross-sectional morphologies of the prepared membranes were analyzed by scanning electron microscopy (SEM-mask-Hitachi 8220). The adsorption property of the ZSM-5 was measured as described in this study. We made three solutions of different concentrations, i.e., 0.5 mmol L^−1^, 1 mmol L^−1^, and 10 mmol L^−1^, containing equimolar of LiCl and MgCl_2_, respectively. Then, 2 g of dried ZSM-5 (H-form) was added to each solution (30 mL) and stirred for a certain time (one day and six days) at room temperature. The solutions were centrifuged at 10,000 rpm for 5 min, and the supernatant (2 mL) was collected from each solution and tested by inductively coupled plasma atomic emission spectrometer (ICP-AES, Optima 7300 DV, Waltham, MA, USA) for Li^+^ and Mg^2+^ ions, respectively. The crystallinity of the ZSM-5 zeolite powder was analyzed by X-ray diffractions (XRD). The surface aperture analyzer was used to obtain the surface area and pore size distribution of the commercial ZSM-5 zeolite.

Water uptake (WU) and area swelling were measured according to the reported literature [40]. First, the membrane samples (1.5 × 1.5 cm^2^) were dipped in DI water for 24 h to fully hydrate and weigh as W_wet_. The samples were then dried in an oven at 60 °C and reweighed as W_dry_. The WU was calculated according to the difference in weight using Equation (1). Similarly, the area of the dried (A_dried_) and wet (A_wet_) membrane samples were measured and the area swelling can be calculated using Equation (2), as given below.

(1) $$Water\;uptake\;(\%)=\frac{W_{wet}-W_{dry}}{W_{dry}}\text{×}100$$

(2) $$Area\;swelling\;(\%\;)=\frac{A_{wet}-A_{dry}}{A_{dry}}\text{×}100$$

### 2.4. Current-Voltage (I-V) Curves

**Figure 1:**
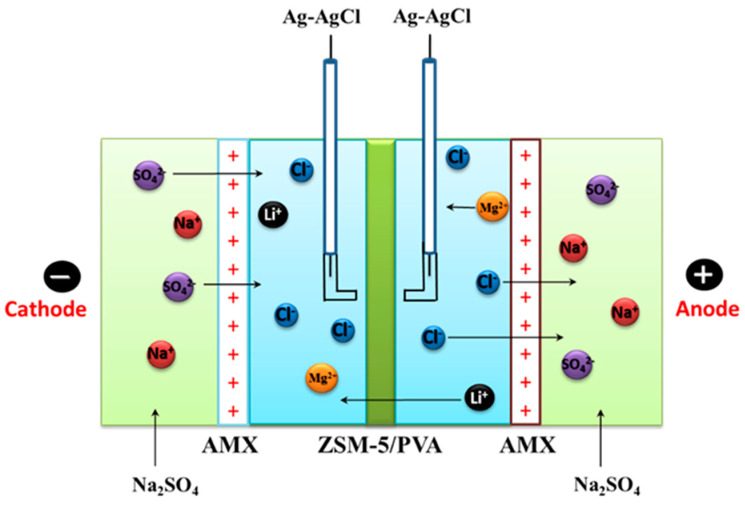
Schematic diagram of the testing device for the I–V curve.

The I-V curves of ZSM-5/PVA-based MMMs were obtained by using a device with a four-compartment cell as in Figure 1 with an exposed membrane’s area of 7.07 cm^2^. The anode and cathode chambers (filled with 0.3 mol L^−1^ Na_2_SO_4_ solution) are separated from the middle two chambers by a couple of membranes, i.e., AMX. The testing membrane was placed in the middle of AMX membranes separating diluted and concentrated chambers. The diluted chamber and the concentrated chamber were filled with the same mixed solution of 0.1 mol L^−1^ LiCl and 0.1 mol L^−1^ MgCl_2_, respectively. A direct current (DC) power supply (WYL1703, Hangzhou Siling Electrical Instrument Ltd., Hangzhou, China) was connected with a pair of Pt electrodes on either side, and the current density was increased steadily from 0 to 100 mA cm^−2^, while the difference in potential was recorded using a multimeter (Victor Hi-Tech Co., Ltd., VC890C^+^, Shenzhen, China) attached with a couple of Ag-AgCl reference electrodes near the membrane surface. Before the test, the membranes were equilibrated in the mixed solution of 0.1 mol L^−1^ LiCl and 0.1 mol L^−1^ MgCl_2_ for 24 h. Peristaltic pumps (YZ15, Baoding Lead Fluid Co., Ltd., Baoding, China) were used to circulate the corresponding solutions in their respective chambers.

### 2.5. Evaluation of the Cations Permselectivity

The permselectivity of the prepared membranes with an effective area of 7.07 cm^2^ was investigated using a similar device, as in Figure 1. The diluted chamber was filled with a 100 mL solutions of 0.1 mol L^−1^ LiCl/0.1 mol L^−1^ MgCl_2_ (for Li^+^/Mg^2+^ system) or 0.5 mol L^−1^ H_2_SO_4_/0.23 mol L^−1^ ZnSO_4_ (for H^+^/Zn^2+^ system) mixtures while the concentrated chamber was filled with 200 mL of 0.01 mol L^−1^ KCl solution, respectively. An electrodes’ rinse solution of 0.3 mol L^−1^ Na_2_SO_4_ was circulated in the electrode’s chambers. The solutions were circulated with a pair of peristaltic pumps at the flow rate of 5.2 L h^−1^ to avoid the concentration polarization. Two auxiliary membranes (AMX) were used on either side of the testing membrane to complete the ED setup. During the ED experiments, a DC power was used to provide a constant current density of 30 mA cm^−2^. The samples collected (from the concentrated section) after 1 h, were tested for Li^+^ and Mg^2+^ ions by ICP-AES, while the H^+^ ion centration was analyzed by acid-base titration using phenolphthalein as an indicator. The membrane’s permselectivity was calculated using Equation (3) as follows.

(3) $$J=\frac{\left(C_{t}-C_{0}\right).V}{A_{m}.t}$$

where J (mol cm^−2^ s^−1^) is the cationic flux while $c_{t}$ (mol L^−1^) and $c_{0}$ (mol L^−1^) represent the cation concentrations in the concentrated chamber at time t and 0, respectively. V (dm^3^) is the volume of the concentrated solution. $A_{m}$ means the effective area of the tested membrane.

The perm-selectivity ($P_{M^{2+}}^{N^{+}}$) between monovalent and divalent cations was calculated as reported [41].

(4) $$P_{M^{2+}}^{N^{+}}=\frac{J_{N^{+}}}{J_{M^{2+}}}\frac{C_{M^{+2}}}{C_{N^{+}}}$$

In Equation (4),$\;J_{N^{+}}$ and $\;J_{M^{2+}}$ are the fluxes of monovalent and divalent cations, whereas $c_{N^{+}}$ (mol L^−1^) and $c_{M^{2+}}$ (mol L^−1^) are the average molar concentrations of the monovalent and divalent cations in the diluted chamber during the experiment, respectively.

## 3. Results and Discussion

### 3.1. Surface Area and Pore Size Distribution Analysis of the ZSM-5

**Figure 2:**
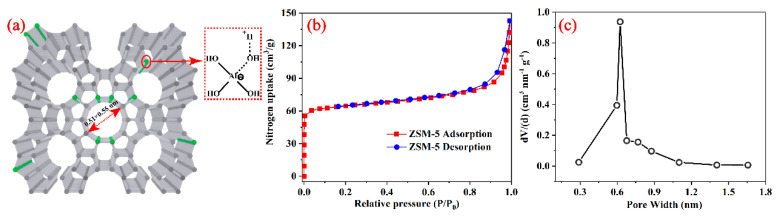
(**a**) Skeletal diagram, (**b**) nitrogen adsorption-desorption isotherms, and (**c**) pore size distribution profiles of ZSM-5 zeolite.

To characterize the microporous structure of the ZSM-5 zeolite, the nitrogen adsorption-desorption isotherm was measured at 77 K. As shown in Figure 2, the result of isotherm sorption profiles showed a typical type I curve (Figure 2b), which is following the IUPAC classifications and indicates the microporous characteristics of the ZSM-5 material. The Brunauer–Emmett–Teller (BET) surface area was calculated to be 288 m^2^ g^−1^, and the pore size distribution mainly focused on a 0.6 nm (Figure 2c) in agreement with that of the skeletal diagram of ZSM-5 zeolite (Figure 2a). The pore size is suitable for ions separation based on size-selective sieving.

### 3.2. Adsorption Capacity of ZSM-5

**Figure 3:**
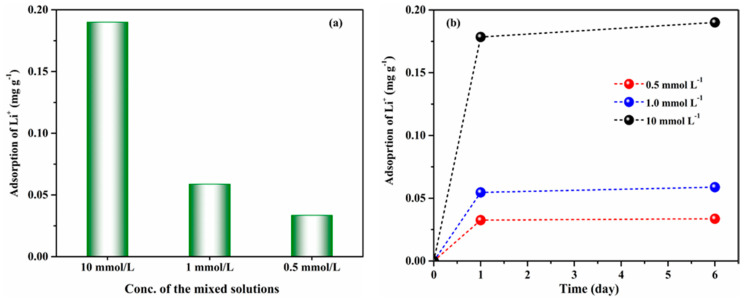
The adsorption property of ZSM-5 for LiCl/MgCl_2_ mixed solutions: (**a**) Li^+^ adsorption vs. concentration of solution, (**b**) Li^+^ adsorption vs. time (day), respectively.

The adsorption capability of the pristine ZSM-5 was evaluated for various concentrations of the binary solution of LiCl/MgCl_2_ at 25 °C for a different interval of time, as given in Figure 3. The acquired results revealed that ZSM-5 is very selective for the monovalent cation as compared with the divalent cation. For instance, when a high concentration (10 mmol L^−1^ LiCl/MgCl_2_) solution was used, the adsorption capacity of ZSM-5 for Li^+^ ion was extraordinary, while the Mg^2+^ did not show noticeable adsorption and we did not include it in Figure 3. When the solution concentration was decreased to 1 and 0.5 mmol L^−1^, the adsorption capacity further decreased. The adsorption ability of the ZSM-5 can be explained by considering the ion-exchange reaction between the Li^+^ in the solution and H^+^ of the ZSM-5 material, i.e., H^+^ is replaced with Li^+^ more quickly as compared with Mg^2+^ ions due to size exclusion and less solvation effect. For a different interval of time (Figure 3b), the initial adsorption takes place very promptly, and then the equilibrium is established. Appropriate Li^+^ ions are maintained inside the ZSM-5 framework for charge compensation and no further exchange takes place. The adsorption results indicate that ZSM-5 are suitable to prepare MMMs for monovalent cations separation.

### 3.3. Morphology

**Figure 4:**
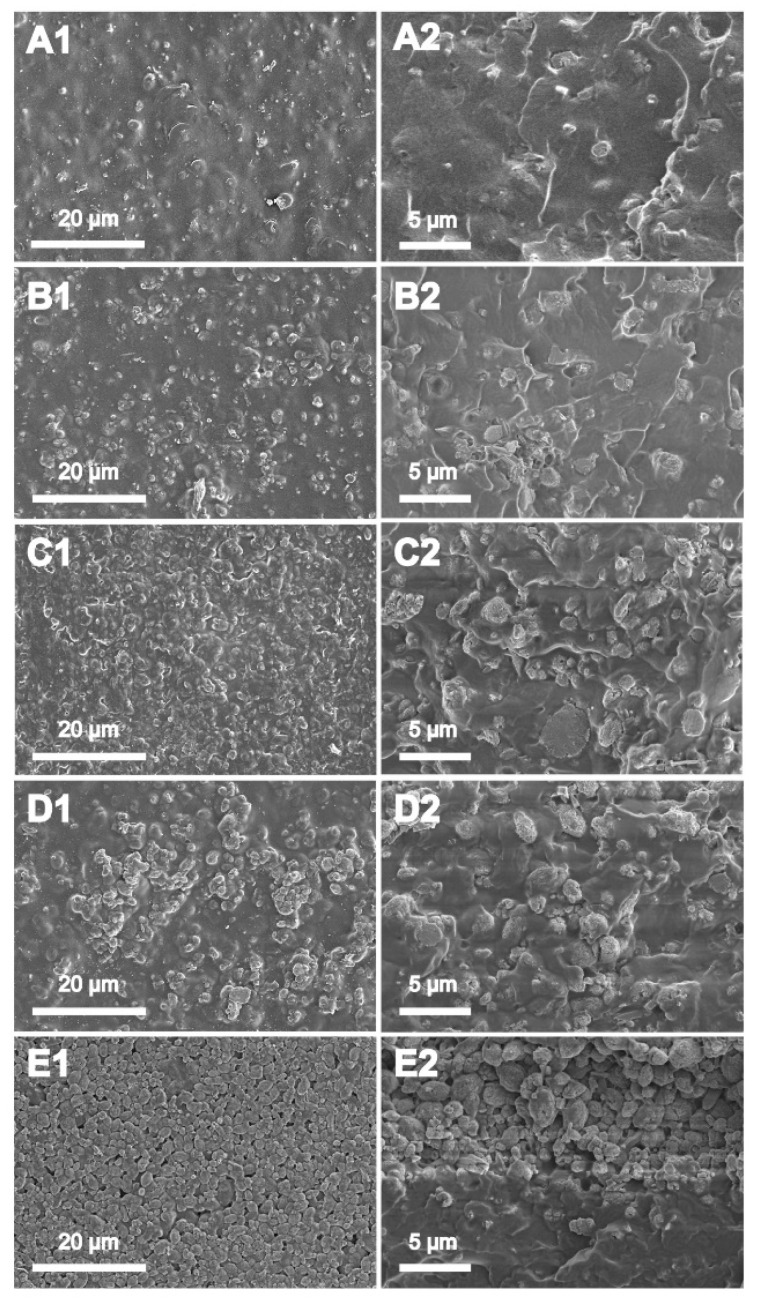
Surface (**A1**–**E1**) and cross-section (**A2**–**E2**) morphologies of ZSM-5/PVA-based mixed matrix membranes (MMMs): (**A**), (**B**), (**C**), (**D**) and (**E**) are 20 wt%, 30 wt%, 40 wt%, 50 wt%, and 60 wt% zeolite doping content, respectively.

The uniform dispersion of the ZSM-5 was examined using SEM for the representative membranes, as given in Figure 4. The surfaces were assigned as A1, B1, C1, D1, and E1, while the cross-sections were nominated as A2, B2, C2, D2, and E2, respectively. The SEM micrographs reveal that, when the concentration of ZSM-5 was 20 wt%, particles were smaller in dimensions with a consistently disseminated PVA profile. For a minute quantity of ZSM-5, the PVA substrate quickly established physical interaction with the ZSM-5 particles via hydrogen bonding between the –OH group of the PVA and oxygen of the doping material [42]. However, when the quantity of ZSM-5 expanded to a maximum of 60%, the particles lump into agglomerates and are hard to scatter uniformly in the PVA solution, as can be seen in Figure 4E. These lumps are more visible in the cross-section analysis of the membranes. The disturbance of the PVA crystal region (explained in the XRD section) and relaxation of the PVA chains may improve ionic fluxes during the ED process. Thus, it is indispensable to choose the optimal quantity of the ZSM-5 to thoroughly adjust it in the membrane’s configuration for desired homogenous membranes.

### 3.4. XRD Analysis

**Figure 5:**
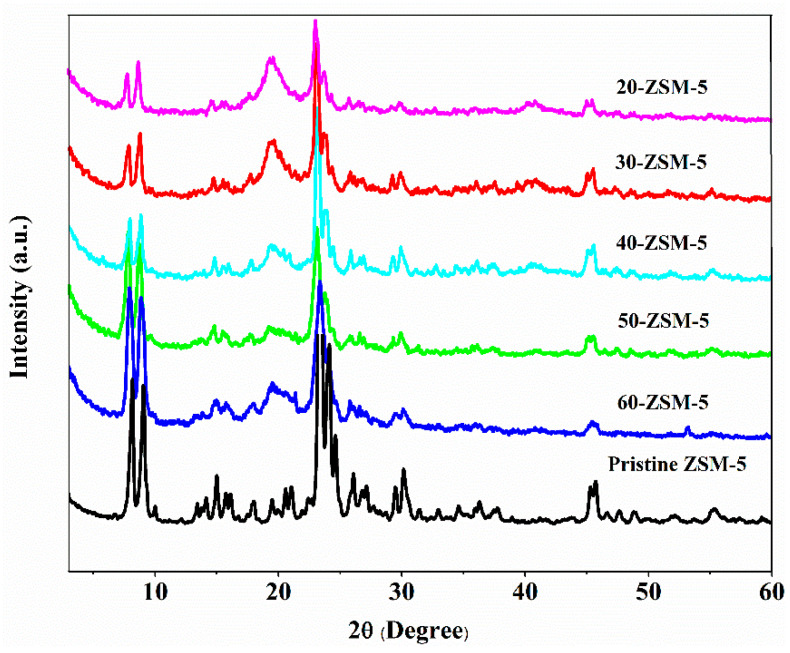
XRD pattern of ZSM-5/PVA-based mixed matrix membranes (MMMs) in comparison with the ZSM-5 zeolite powder.

The ZSM-5 is a microporous crystalline material, as discussed in the earlier section. It was assumed that, during membranes’ synthesis, the fabrication process might disrupt its indigenous characteristics, which are crucial for selective cations separation. The powder X-ray diffraction (PXRD) pattern of the pristine ZSM-5 was compared to the ZSM-5 modified membranes, which are given in Figure 5. The modified ZSM-5 membranes demonstrate the identical characteristic peaks as in pristine ZSM-5 at 2θ at 6°–10° (doublet), and 22°–25° (triplet), respectively, which indicated that the crystalline structure of ZSM-5 was not altered after incorporation in the PVA backbone [43]. Moreover, the crystallinity of the PVA (at 2θ of 19.4°) decreased when the dosage of ZSM-5 improved from 20% to 60% [44]. It might be due to the breaking of hydrogen bonds between PVA chains and the insertion of ZSM-5 as an intervening material. Additionally, when the ZSM-5 loading was adequately high (60%), the aggregate of the ZSM-5 nanoparticles may form (SEM section) and, consequently, lower the crystalline behavior of the PVA profile. In conclusion, the XRD pattern of the ZSM-5 doped membranes have no noticeable difference with the original ZSM-5, which justify the synthesis protocol for the high permselective membranes.

### 3.5. Water Uptake (WU) and Area Swelling

**Figure 6:**
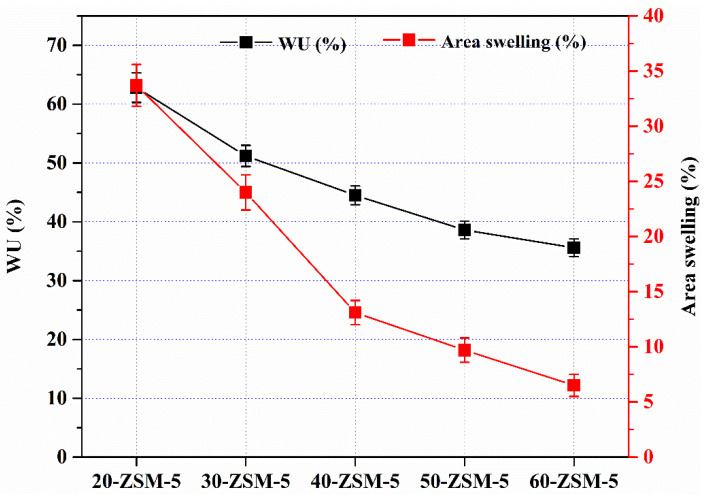
Water uptake (WU) and area swelling of the prepared MMMs.

The physical characteristics such as water uptake (WU) and area swelling of membranes are essential parameters that appraise the membrane applicability for the purpose applications. This primarily occurs when the PVA substrate is employed for membrane synthesis. In the present case, the modification of PVA with ZSM-5 has acknowledged in depreciating its swelling behavior in the water-based application. Comprehensive articles have been reported in this regard [45,46,47,48]. The WU and area swelling results are assessed as given in Figure 6. After alteration with ZSM-5, the WU and area swelling were reduced to 62.8% and 33.7%, respectively. Further raising the amount of ZSM-5 to 60%, both parameters were significantly lowered to 35% and 6.5%, respectively. The decrease in WU and area swelling could be attributed to the engagement of the -OH of the PVA with oxygen sites in the zeolite via hydrogen bonding. For high permselectivity, high membranes WU and area swelling are discouraged to evade the leakage of more hydrophilic cations such as Mg^2+^, Zn^2+^, and structural deformation during water-based applications. From Figure 6, it declares that ZSM-5 modified membranes exhibited lower WU and area swellings since we increased the quantity of ZSM-5. It may presume that the interfacial hindrance interaction was lessened and chain motion of the base membrane was restricted, which was the principal theme of ZSM-5 modified membranes. Hence, the developed membranes, particularly 60%, demonstrated considerably lower WU and area swelling than the rest of the membranes due to the more potent inhibition. The membrane’s surface inhibition is beneficial to segregate ions on the base of their hydrophilic nature. Additionally, it can be realized that the reduced area swelling may also improve the structural stability of ZSM-5/PVA-based membranes.

### 3.6. Current-Voltage (I–V) Curves

**Figure 7:**
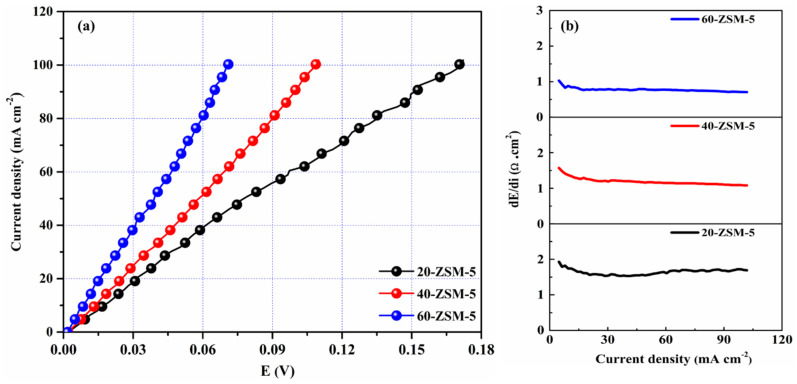
(**a**) Current-voltage curves, and (**b**) membrane resistance of representative’s membranes.

The current–voltage investigation is an essential tool to explain the behavior of IEMs over a range of current applications. Figure 7 exhibited the I-V curves for the ZSM-5 modified membranes. The membrane resistance (R_M_) was obtained by plotting the ratio of dE/di vs. current density, as given in Figure 7b, which demonstrated the decrease of membranes’ resistance by inserting ZSM-5 in the PVA backbone. This behavior was expected since the cation exchange carriers were introduced with the ZSM-5. For instance, when the ZSM-5 content was increased from 20 to 60 wt%, the R_M_ decreased from 1.64 Ω·cm^2^ to 0.77 Ω·cm^2^. This decrease in resistance can be associated with the structure of the ZSM-5 in the membranes. The 20-ZSM-5 membrane with the lowest content does not have adequate cations ways, and the substrate provides a high impedance to the incoming cations. However, when the dosage of ZSM-5 was further increased, the R_M_ was significantly lowered due to the generation of more channels to predominate cations’ transportation. The optimized content plus compactness of the membranes are the critical factors conferring monovalent cations’ perm-selectivity to these membranes (see ED section).

Notably, the developed membranes unveil high limiting current density beyond 100 mA cm^−2^. The high limiting current density is affiliated with the foundation of nanochannels (nano porosity was confirmed in the BET). Moreover, the lowest R_M_ value calculated for the modified membranes (0.77 Ω·cm^2^ for 60-ZSM-5 membrane) is even lower as compared with the reported membranes [49,50]. As asserted earlier, the reduction in the R_M_ with the dosage of ZSM-5 can be considered a supplementary sign of the successful establishment of ion transport channels in the membrane profile. From the perspective of I-V curves, the MMMs have high limiting current density and low R_M_, which indicated more applicability and flexibility of the developed membranes for practical applications.

### 3.7. Electrodialysis (ED) Experiments

**Figure 8:**
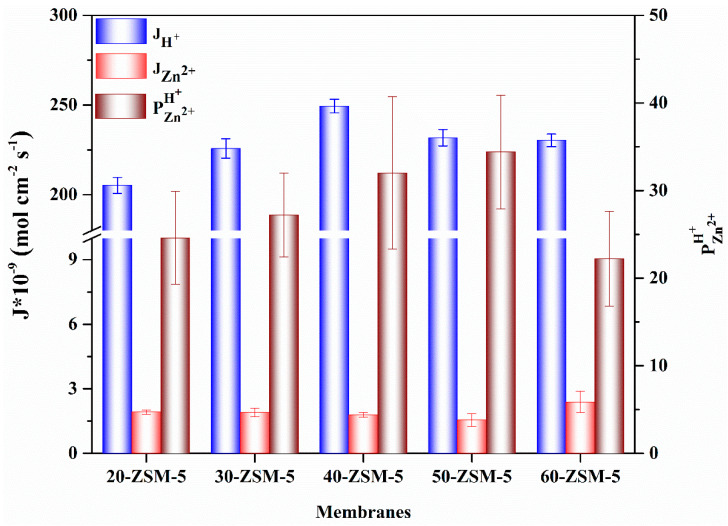
The ion flux and perm-selectivity of ZSM-5/PVA-based MMMs for H^+^/Zn^2+^.

**Figure 9:**
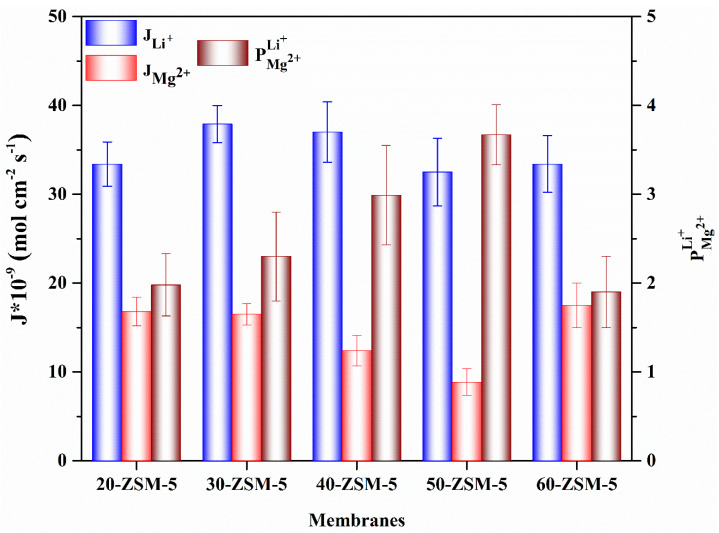
The ion flux and perm-selectivity of ZSM-5/PVA-based MMMs for Li^+^/Mg^2+^.

The membranes’ performance with various concentrations of the ZSM-5 was inquired for a pair of mixed feed solutions such as 0.1 mol L^−1^ LiCl/0.1 mol L^−1^ MgCl_2_ and 0.5 mol L^−1^ H_2_SO_4_/0.23 mol L^−1^ ZnSO_4_, respectively. The ion fluxes and perm-selectivity for both systems were conducted at 30 mA cm^−2^ current density and 25 °C, as represented in Figure 8 and Figure 9, respectively.

Figure 8 depicts the ions fluxes and perm-selectivity of the modified membranes in a mixture solution (H^+^/Zn^2+^ system). The low applied current density (30 mA cm^−2^) was chosen to avoid the limiting current density’s complications. In these experiments, we did not test the PVA base membrane due to the high membrane resistance and no noticeable perm-selectivity. Supplementing the membrane pattern with 20% of ZSM-5, the H^+^ flux was approached to 2.05 × 10^−7^ mol cm^−2^ s^−1^, while the Zn^2+^ flux was marked to 1.92 × 10^−9^ mol cm^−2^ s^−1^ and the perm-selectivity was recorded up to 24.6. The high ion flux and perm-selectivity of the ZSM-5 modified membranes could be entirely ascribed to the ZSM-5, which contributed ion-exchange localities to the membrane profile. When the ZSM-5 amount was increased from 20% to 40%, the H^+^ flux was increased, i.e., 2.49 × 10^−7^ mol cm^−2^ s^−1^, while the Zn^2+^ flux was dropped from 1.92 × 10^−9^ mol cm^−2^ s^−1^ to 1.72 × 10^−9^ mol cm^−2^ s^−1^ and the perm-selectivity was further improved to 32.

The H^+^ with the lowest hydrated radius of less than 0.24 nm can efficiently progress through the porous ZSM-5 materials with a pore diameter of about 0.6 nm. Zn^2+^ has high charge density and sustains a larger hydrated radius of 0.6 nm and were screened-out due to the size exclusion (SE) effect. When the ZSM-5 was further increased to 50%, both cations fluxes (H^+^ flux is less decreased) were decreased because of the more compact structure of membranes. However, the perm-selectivity was increased to 34.4. By, subsequently, increasing the ZSM-5 contents up to 60%, the perm-selectivity was dropped to 22 due to the ineffective adhesion among the PVA and ZSM-5, which produced non-selective voids at the ZSM-5/polymer interface (SEM results) and decreased the membrane perm-selectivity.

We also explored the performance of membranes for the Li^+^/Mg^2+^ system using the same operational conditions (30 mA cm^−2^). The adsorption characteristics of the ZSM-5 material exhibited high adsorption selectivity to the monovalent cations. Hence, it is essential to screen-out the divalent cations using the ZSM-5 in the membrane materials for particular applications. As shown in Figure 9, the Li^+^ ion flux progressed to 3.34 × 10^−8^ mol cm^−2^ s^−1^, while the lower flux of Mg^2+^, i.e., 1.68 × 10^−8^ mol cm^−2^ s^−1^, rationalized the rejection property of membranes (perm-selectivity = 1.98). Likewise, by increasing the quantity of ZSM-5 up to 50% (50-ZSM-5), one can notice insignificant shrinkage in the flux of the monovalent cation, while the flux of divalent cations drastically descended to 8 × 10^−9^ mol cm^−2^ s^−1^ and delivered high perm-selectivity of 3.7. However, further expanding the value of ZSM-5 was not effective, and may create membranes with defects and degrade the perm-selectivity. The high flux of monovalent cations can be characterized by ion size exclusion and the selective replacement of H^+^ with Li^+^ ions in comparison with the Mg^2+^ ion, as demonstrated earlier in the adsorption part. The H^+^ can quickly get through the pores due to the smaller hydrated Stokes radius to obtain high flux. However, the Li^+^ possesses a lower hydration number and hydration energy (∆G_hyd_^0^), i.e., 5.2 and −475 kJ mol^−1^ as compared with the Mg^2+^ (10.0, -1830 kJ mol^−1^) [51]. Therefore, the Li^+^ can easily de-hydrate to permeate across the pores of ZSM-5.

In view of the ED results, ZSM-5/PVA membranes performed well in both acid recovery and Li+ recovery, respectively. The achieved results are encouraging when compared with the commercial (CSO) and literature reported membranes, as listed in Table 1. The satisfactory performance of prepared MMMs can be accredited to the cationic exchange sites and crystalline structure. Our membranes exhibited high limiting current density, which is profitable for industrial applications.

## 4. Conclusions

In the present research, the impact of the ZSM-5 zeolite-based membranes on the selective removal of monovalent cations was investigated. For this purpose, we preferred a PVA polymer as a substrate and then consolidated various amounts of ZSM-5 using an economical design to prepare permselective membranes. Initially, the adsorption for the monovalent cation, porosity by BET, and crystalline feature by XRD were exclusively scrutinized, which validated the usage of the ZSM-5 for membranes application and ions separation. The choice of the PVA polymer was productive to establish a strong interaction with the ZSM-5 and to provide excellent dispersion media. Moreover, the SEM micrographs revealed an excellent dispersion of the ZSM-5 by creating permeation pathways without any aggregation for a controlled quantity. The controlled amount of ZSM-5 (40-ZSM-5 and 50-ZSM-5) produced a significant enhancement in both monovalent cation permeability and perm-selectivity. The physiochemical characteristics such as WU, area swelling, and membrane resistance results revealed that the ZSM-5 doping has a substantial impact on desired properties. The MMMs exhibited very high limiting current density, which indicates more applicability and flexibility in the practical application. The conferred strategy is very adaptable for the synthesis of MMMs for a wide variety of zeolites and polymer combinations for envisioned purposes.

## Figures and Tables

**Table 1:** Characteristics’ properties of recently reported membranes and ZSM-5/PVA-based MMMs.

Membranes	Morphology	Systems	Perm-Selectivity	[Ref.]
CSO	dense	H^+^/Zn^2+^, Li^+^/Mg^2+^	3.5, 1.6	[40,52]
SBQAPPO	dense	H^+^/Zn^2+^, Na^+^/Mg^2+^	23.5, 7.4	[52]
Neosepta CMX	dense	Na^+^/Mg^2+^	1.6	[41]
Asymmetric porous	porous	Na^+^/Mg^2+^	3.3	[2]
DL-2540 NF	porous	Li^+^/Mg^2+^	3.3	[20]
ZSM-5/PVA-based MMMs	porous	H^+^/Zn^2+^, Li^+^/Mg^2+^	34.4, 3.7	This work
